# INVOLVEMENT IN CARE DECISION-MAKING AND ADVERSE OUTCOME ONSET IN COMMUNITY-DWELLING CARE RECIPIENTS IN JAPAN

**DOI:** 10.1093/geroni/igac059.2563

**Published:** 2022-12-20

**Authors:** Ayane Komatsu, Takeshi Nakagawa, Taiji Noguchi, Tami Saito

**Affiliations:** National Center for Geriatrics and Gerontology, Obu, Aichi, Japan; National Center for Geriatrics and Gerontology, Obu, Aichi, Japan; National Center for Geriatrics and Gerontology, Obu, Aichi, Japan; National Center for Geriatrics and Gerontology, Obu, Aichi, Japan

## Abstract

The involvement of older adults in care decision-making may enhance their daily life motivation and quality of life. Furthermore, it could contribute to their better prognosis in long-term care. We examined the association between decision-making involvement and the onset of adverse outcomes, such as institutionalization and death, among older adults under long-term care. This study used two-year longitudinal survey data of Japanese community-dwelling care recipients aged 65 and above. The participants were followed regarding the onset of institutionalization and deaths. The status of involvement in decision-making was assessed based on one item and the selection among the following response options: “very much involved,” “fairly involved,” “not very involved,” “never involved,” “unclarified wishes,” and “absence of person supporting decision-making.” A multivariable logistic regression analysis estimated the odds ratios (OR) and 95% confidence intervals (CI) for the onset of adverse outcomes, composite of institutionalization and death. A total of 707 participants with no severe cognition disabilities (MMSE>12) and no missing variables at the baseline were included and responded to the follow-up survey. At the baseline, 36.5% reported being very much involved in decision-making. The onset of adverse outcomes was observed in 17.5% of participants (institutionalization, 5.1%; death, 12.4%). Compared to those with very high involvement in decision making, those who were not involved were more likely to have adverse events, even after adjusting for covariates (OR=2.86 [95% CI: 1.21-6.76], p=0.016). Our findings show the importance of decision-making involvement in daily care regarding better prognoses in long-term care.

